# Spontaneous Endoscopic Esophageal Stent Fracture Post-endoscopic Placement: A Case Report

**DOI:** 10.7759/cureus.49406

**Published:** 2023-11-25

**Authors:** Zoha Zahid Fazal, Muhammad Bilal Ibrahim, Muhammad Ibrahim Saeed, Syedda Ayesha, Atif Majeed

**Affiliations:** 1 Medical College, Aga Khan University, Karachi, PAK; 2 Internal Medicine, John H. Stroger Cook Country Hospital, Chicago, USA; 3 Gastroenterology and Hepatology, Aga Khan University Hospital, Karachi, PAK; 4 Gastroenterology, Aga Khan University Hospital, Karachi, PAK

**Keywords:** distal displacement, stent fracture, fully covered metallic stent, esophagogastroduodenoscopy, esophageal perforation

## Abstract

Endoscopic esophageal stent (EES) placement is an important tool for the non-operative management of esophageal pathologies. A rare and infrequently reported complication of EES placement is stent fracture and subsequent migration of the broken fragments. We report a rare case of a spontaneous EES fracture from Pakistan four weeks following its placement for esophageal perforation management, and an uneventful endoscopic retrieval of the fractured stent pieces. The recommended guidelines from available, albeit limited, research literature are also discussed as part of this case report.

## Introduction

Endoscopic esophageal stent (EES) placement is commonly performed to manage refractory esophageal strictures, iatrogenic esophageal perforations, postoperative leaks, tracheoesophageal fistulas, and for the palliative management of esophageal cancer [1]. The growing preference of stenting over operative repair as a therapeutic option for esophageal perforation is often attributed to its less invasive nature [2]. Endoluminal stent placement instills prompt closure of the patent perforation site, prevents the need for an urgent thoracotomy, and eliminates the soilage of any thoracoabdominal cavity [3].

Metal stents may be made of stainless steel, nickel, and/or titanium, and are an improved upgrade from Celestin tubes to manage refractory esophageal obstruction [4] due to their easier and safer deployment, higher biocompatibility, and increased malleability [5]. However, metallic stents may be exposed to significant stress-induced damage which may eventually weaken the metal structure leading to subsequent fracture and fragmentation.

Esophageal stents that are currently preferred feature self-expanding metal stents (SEMS), SEMS with an anti-reflux valve, self-expanding plastic stents, drug-eluting and radioactive SEMS, and biodegradable stents [6]. While fully covered stents with an outer synthetic coating of silicone or polyurethane derivatives offer greater biocompatibility, a partially covered SEMS has bare metal at its ends to allow embedding into the esophageal wall and prevent stent migration [7]. However, a recent meta-analysis found no significant difference in stent migration with fully or partially covered SEMS when used for the palliative treatment of esophageal malignancy. The removal of esophageal stents is generally recommended four to six weeks after their initial placement [8].

## Case presentation

**Figure 1:**
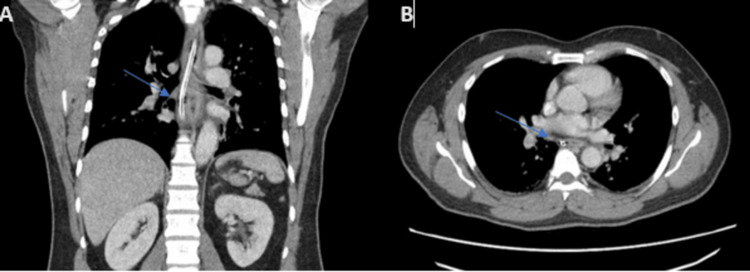
(A) Axial section CT image showing a nasogastric tube passed under the endoscopic vision across the perforated area at the level of thoracic vertebra. (B) Coronal section CT image with contrast showing a small linear contained leak draining into a small walled-off collection measuring approximately 24 x 9 mm, to the left of esophagus (arrow).

A 51-year-old gentleman with no known comorbidity presented to us with a history of chest pain and odynophagia following the ingestion of apricot with seed. His past medical history was significant for an episode of food bolus obstruction 11 years back for which he underwent an uneventful esophagogastroduodenoscopy (EGD) to remove the lodged food obstructing the esophagus. On arrival in the emergency department, the clinical examination was unremarkable. The patient was advised to have a chest X-ray (CXR) in anteroposterior view which was normal. A chest computed tomography (CT) scan with contrast was suggestive of an esophageal perforation with localized collection to the left side of subcarinal esophagus (Figure 1).

**Figure 2:**
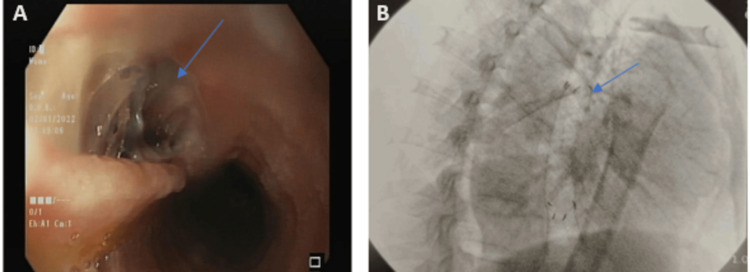
Endoscopic image showing full thickness perforation (arrow) starting from 30 cm and extending till 33 cm relative to the incisors in the esophagus. (B) Fluoroscopic image showing fully covered self-expandable metallic stent (arrow) post-procedure.

Cardiothoracic and General Surgery teams were taken on board, and they advised to proceed with endoscopic esophageal stent placement. The patient underwent an EGD in the operating room under conscious sedation and aseptic protocol. EGD findings showed a full-thickness linear perforation of approximately 3cm in the distal esophagus (Figure 2A). A fully covered self-expandable metallic esophageal stent (Bonastent; Thoracent, Huntington, NY, USA) of 20 mm x 100 mm dimensions, was placed via endoscopic guidance (Figure 2B). The patient remained stable post-procedure, tolerated his diet well, and hence, was discharged after 24 hours of observation.

**Figure 3:**
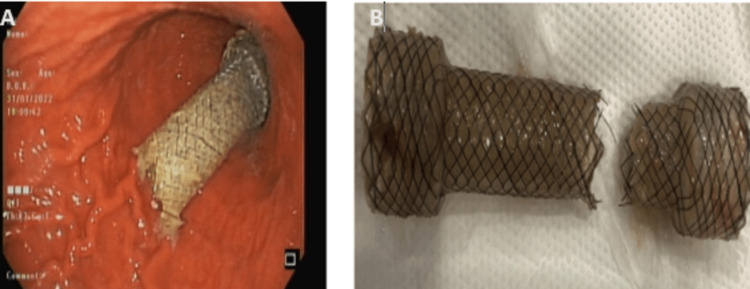
(A) Endoscopic image showing fractured distal part of the self-expanding metal stent (SEMS) in the stomach. (B) Complete circumferential fracture of SEMS.

Thereafter, the patient was electively admitted for stent removal four weeks after its placement. A CT scan chest showed fractured stent with its distal part migrated to stomach and healed previously perforated esophagus. He underwent a successful EGD procedure, and the stent was visualized on endoscopy. The previously placed esophageal stent was circumferentially broken, the proximal part was excised from the lower esophagus while the distal stent fragment had migrated to and was snared from the stomach (Figure 3). After removal of the stent pieces, the previous perforation site was examined through fluoroscopic imaging which had successfully healed.

## Discussion

While stent placement is often safe, several complications have been recognized, including stent fracture, migration, and occlusion [5]. Involvement of the gastrointestinal tract can potentially lead to fistulation, hemorrhage, stricture formation, obstruction, perforation, and reflux. Symptoms may include chest pain, food bolus impaction, aspiration, and hematemesis. Stent fracture and the consequent migration of the stent fragments are the most common complication among all, with incidental frequency ranging from 7% to 75% as described in multiple case reports, dating as early as 1999 and reported periodically since then [4].

The potential complications reported following successful EES deployment include reactive stenosis, pressure-induced ischemia, ulceration, perforation, bleeding, and failure of device retrieval [9]. EES fracture is a rather rare complication, commonly reported eight to 40 weeks after EES placement [10].

Commonly reported cases of EES fractures involved their placement for the management of malignant obstruction of the gastric outlet, biliary tract, duodenal ampulla, and colon [4]. Both partial and complete circumferential stent fractures of enteral metallic SEMS have been reported. The probability of stent migration depends on the type, diameter, coverage, and precise location of placement. As per literature, up to 20% of covered stents migrate from the esophageal gastric junction [11]. Additionally, most stents do not get displaced beyond the stomach and have low associated morbidity with zero reported mortality to date [12].

Possible factors accounting for stent fracture have been comprehensively discussed. The use of balloon catheters to dilate esophageal metal stents for rapid and maximal stent expansion as well as laser application to control bleeding from tumor ingrowths have been associated with stent fracture [13]. Studies have postulated that thermal straining of the metal can possibly result in stent fracture [14] while high-dose radiation therapy has been to shown to be associated with metallic tracheobronchial stent fractures in one case series [15].

Different management strategies have been attempted for retrieval of fractured stent fragments. Most cases with migrated stents can be managed by non-operative management, endoscopic removal, spontaneous fecal release, or uncomplicated retention in the body [11]. Placement of a second stent may be needed for the palliative and symptomatic management of pre-existent esophageal pathologies. While radiologic intervention or surgical management of stent migration is an uncommon occurrence, operative techniques may involve laparotomy incision, enterotomy for stent retrieval, bowel resection, and primary anastomosis [11].

## Conclusions

Endoscopic stenting remains an important non-surgical modality for esophageal neoplasia. Stent fracture should be a differential for patients presenting with dysphagia and obstructive symptoms following EES placement. Endoscopic therapy and rarely surgery are the recommended management strategies for retrieval of the fragments. Early recognition and care may help prevent complications of endoscopic stent fractures.
